# The radiographic outcome after plating for pubic symphysis diastasis: does it matter clinically?

**DOI:** 10.1007/s00402-022-04411-7

**Published:** 2022-03-12

**Authors:** Kuo-Yuan Tseng, Kai-Cheng Lin, Shan-Wei Yang

**Affiliations:** Department of Orthopedics, Kaohsiung Veterans General Hospital, 386 Ta-Chung 1st Road, Kaohsiung, 81346 Taiwan

**Keywords:** Pubic symphysis diastasis, Plating, Implant failure, Functional outcome

## Abstract

**Introduction:**

Open reduction and internal fixation with plates is the most widespread surgery in traumatic pubic symphysis diastasis. However, implant failure or recurrent diastasis was commonly observed during follow-up. The aim of our study was to evaluate the radiologic findings and clinical outcomes.

**Materials and methods:**

Sixty-five patients with traumatic pubic symphysis diastasis treated with plating between 2008 and 2019 were retrospectively reviewed. The exclusion criteria were a history of malignancy and age under 20 years. Radiographic outcomes were determined by radiograph findings, including pubic symphysis distance (PSD) and implant failure. Clinical outcomes were assessed according to the Majeed score at the final follow-up.

**Results:**

Twenty-eight patients were finally included. Nine patients (32%) experienced implant failure, including four (14%) with screw loosening and five (18%) with plate breakage. Only one patient underwent revision surgery. Postoperatively, a significant increase in PSD was observed at 3 months and 6 months. Postoperative PSD was not significantly different between patients with single plating and double plating, but it was significantly greater in the implant-failure group than in the non-failure group. The Majeed score was similar between patients with single plating and double plating or between the implant-failure group and the non-failure group. Body mass index, number of plates, age, and initial injured PSD were not significantly different between the implant-failure group and the non-failure group. Only a significant male predominance was observed in the implant-failure group.

**Conclusion:**

A gradual increase in the symphysis distance and a high possibility of implant failure may be the distinguishing features of traumatic pubic symphysis diastasis fixation.

The postoperative symphyseal distance achieved stability after 6 months, even after implant failure. Radiographic outcomes, such as increased symphysis distance, screw loosening, and plate breakage, did not affect clinical functional outcomes.

## Introduction

A pelvic fracture, often resulting from high-energy trauma, is a challenging task for both traumatologists and orthopedic surgeons. Pelvic fractures are associated with high rates of morbidity and always warrant urgent management, including immediate resuscitation, damage control, the use of a pelvic binder, emergency angiographic embolization, pelvic packing, the application of an external fixation or the combination of these treatment options [1, 2].

Anterior pelvic ring disruptions are observed in more than 50% of the patients with pelvic ring injuries [3]. The mechanisms of injury can be classified as anterior–posterior compression (APC), lateral compression (LC), vertical shear (VS), or a combination of these based on the Young-Burgess classification [4]. Although traumatic pubic symphysis diastasis can result from any of these types of injuries, anterior–posterior compression is the most common mechanism [5]. The goals of treating a symphysis diastasis include an appropriate reduction and a stable fixation to provide pelvic ring stability, which can effectively diminish the pain, and permit early mobility [6–8]. Numerous surgical methods have been described for treating pubic symphysis diastasis, including plating, external fixation, tension band wiring, sutures, and percutaneous fixation [6, 9–12]. Previous biomechanical studies have indicated that symphysis plating is effective in restoring the anterior ring stability [13], and it has often been used to treat cases of pubic symphysis diastasis worldwide [6]. Regardless of the various surgical methods, it is not uncommon to observe implant failure or a recurrent diastasis of the pubic symphysis from radiography during the postoperative follow-up. Some studies have presented postoperative hardware failure rates and recurrent diastasis rates ranging from 12% to as high as 75% after surgical fixation of a pubic symphysis diastasis [3, 5, 10–18]. However, a relatively low revision rate has been reported, ranging from 0.7% to 8% [5, 10, 14, 15, 17, 18].

Although several studies have commented on implant failure after treating pubic symphysis disruption, the correlation between the radiological changes and the clinical outcomes is not clear. The aim of this study was retrospective to evaluate the radiographic and functional outcomes of patients with traumatic pubic symphysis diastasis treated with plating and to evaluate any associated risk factors of implant failure.

## Materials and methods

### Study populations

Between January 2008 and December 2019, sixty-five patients with traumatic pubic symphysis disruptions were treated in a regional Level 1 trauma center. The authors retrospectively reviewed and monitored the patients under the approval of the institutional review board (KSVGH21-CT6-10). Patients with a history of malignancy or young adults under 20 years of age were excluded. Nine patients who underwent conservative treatment, four patients who died during hospitalization, three patients with a malignant tumor history, and four patients under 20 years old were excluded. The remaining forty-five patients were treated with surgical plating for pubic symphysis disruption. Seventeen patients were lost to follow-up or lacked complete data. The final study cohort was comprised of twenty-eight patients.

### Procedure

**Fig. 1:**
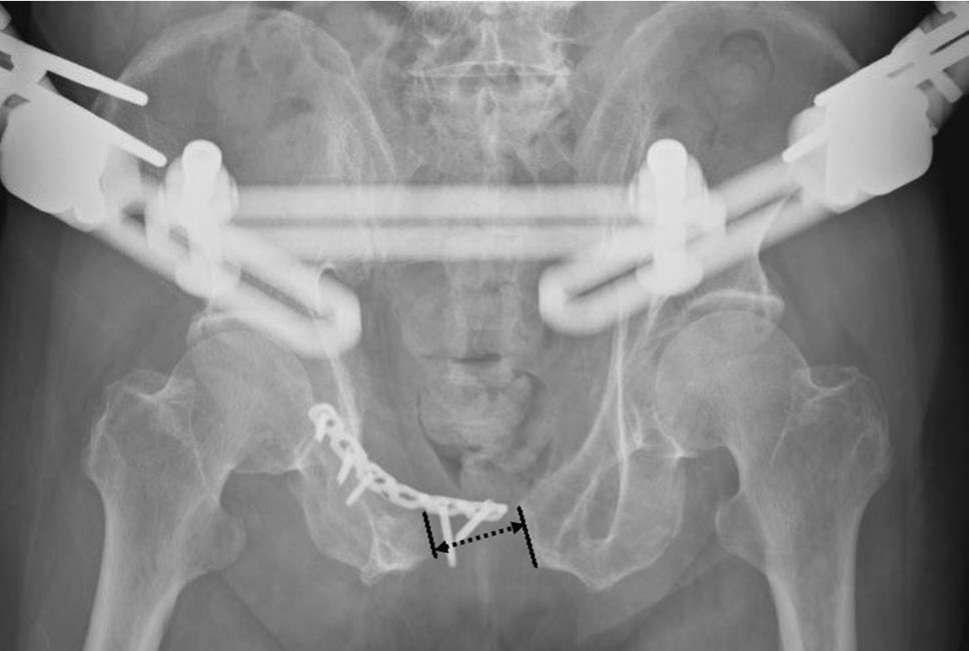
Implant failure with loss of reduction, showing a pubic symphysis distance (arrow dotted line) over 2.5 cm

All injured pelvises were evaluated by radiography and computer tomography. The injured pelvises were classified according to the Young-Burgess Classification. After resuscitation and stabilization of the patient’s vital signs at the time of the initial injury, open reduction and internal fixations were carried out as early as the patient’s general condition allowed. The indication for plating of symphysis diastasis was anterior instability of the pelvic ring. Posterior fixation was additionally performed if posterior instability of the pelvic ring was present. The choice of a single or double plate for the symphysis plating depended on the surgeon’s preference. Rehabilitation interventions were started on the first day after surgery. The patients were encouraged to have early mobilization. Patients with anterior ring disruption alone were allowed partial weight bearing to 50% of their body weight after surgery. Patients with concomitant iliac wing or posterior ring injury were allowed toe-touch weight bearing on the side of hemi-pelvic injury and were allowed to walk on crutches for the first 6 weeks. Partial weight bearing to 50% of the bodyweight was followed during the next 6 weeks. Full weight bearing, as tolerated, commenced 3 months postoperatively. Radiographs of the pelvis anteroposterior (AP) view were obtained at the time of initial injury; immediately postoperatively; and at the 3-, 6-, and 12-month follow-ups. The pubic symphysis distance (PSD) was measured as the narrowest distance between the bilateral pubic ramus on the patients’ radiographs and was recorded (Fig. 1).

### Evaluation of the outcomes

The radiographic outcomes of fixation, including the PSD, plate breakage, and screw loosening, were determined by the radiographic findings. Implant failure was defined as a plate breakage or screw loosening. According to the Young-Burgess classification, a pubic symphysis separation greater than 2.5 cm is unstable, which is a surgical indication. After the surgical reduction and fixation, the PSD will recover with a narrower space. Loss of reduction was defined as a postoperative PSD greater than 2.5 cm during the follow-up, which was suspected to indicate that the pelvic ring was unstable again after being previously stable postoperatively. The clinical functional outcome was assessed according to the Majeed score, which ranged from 0 to 100 [19]. The age, sex, fracture type, body mass index (BMI), and complications of each patient were recorded in detail. All patients were followed up for at least one year after surgery.

### Statistical analysis

The radiographic and clinical results were reviewed by one independent observer. Statistical analyses were conducted using the Statistical Package for the Social Sciences (SPSS) version 22.0 (SPSS Inc., Chicago, USA). The level of significant difference was set at *p* < 0.05.

## Results

Twenty-eight patients, sixteen males and twelve females, with a mean age of 34.3 years (20–81 years), were included in this study. The average follow-up time was 48.6 months (13–76 months). Table 1 presents a summary of the demographic characteristics of the patients.

**Table 1:** Characteristics of Patients

Parameter	Data
Number of patients	28
Sex, male/female	16/12
Mean age (year)	34.3 (20–81)
Fracture type (Young-Burgess classification)	
VS	4 (14.3)
APC-II	11 (39.3)
APC-III	12 (42.9)
LC-II	1 (3.6)
Initial injured PSD (mm)	37.0 (23–115)
Postoperative PSD (mm)	
Immediately	5.3 (2–11)
3 months 6 months	7.9 (3–19)^a^ 8.6 (4–25)^b^
12 months	8.8 (4–26)^c^
No. of implant failures	
Screw loosening	4 (14.3)
Plate breakage	5 (17.9)
No. with loss of reduction	2 (7.2)
No. of secondary operations	1 (3.6)
No of surgical side infections	2 (7.2)
Majeed score at final follow-up	78.8 (59–90)

Continuous data are presented as the mean (range); categorical data are presented as numbers (percentages)

*VS* Vertical Shear, *APC* Anterior–posterior compression, *LC* Lateral compression, *PSD* pubic symphysis distance

*P *values were computed by paired *t* test

^a^*P* < 0.001, compared with immediately postoperative PSD

^b^*P* = 0.019, compared with 3-month PSD

^c^*P* = 0.11, compared with 6-month PSD

### Radiographic outcome

**Fig. 2:**
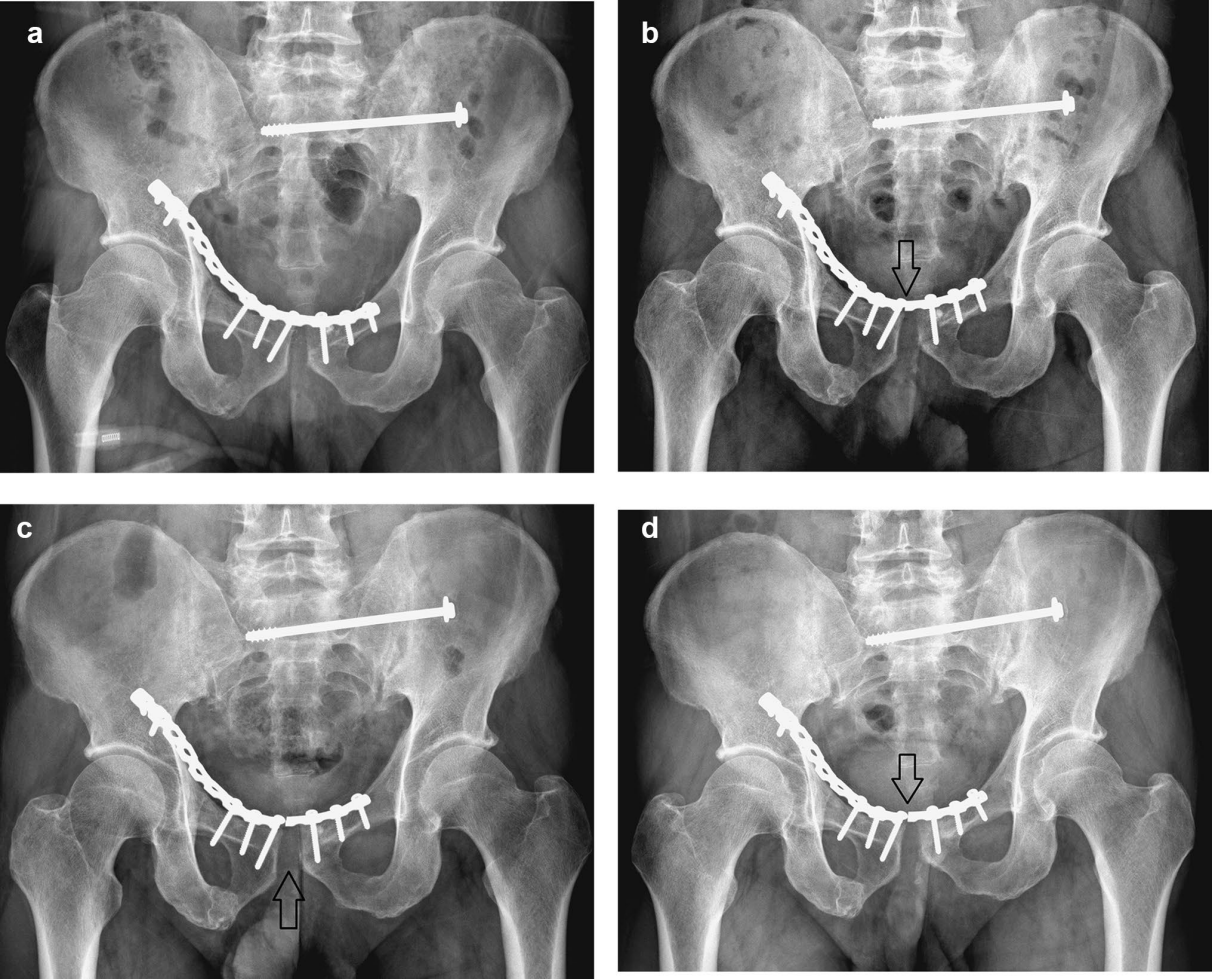
Radiographs of the pelvis in an anteroposterior view after anterior plating and posterior screw fixation. **a** Immediately postoperative. **b** At 3 months, symphysis plate breakage was observed (seen at arrow). **c** At 6 months, the pubic symphysis distance mildly widened (arrow). **d** At 12 months, the pubic symphysis distance became stable without an interval change

Nine patients (32%) experienced implant failure, including four with screw loosening and five with plate breakage during the follow-up. Among the implant failure patients, one patient experienced it immediately during hospitalization, seven had implant failures at 3 months post-operatively, and the remaining patient had implant failure at 6 months postoperatively. Two patients met the criteria of loss of reduction. Only one patient who experienced implant failure immediately during hospitalization underwent a secondary operation for a revision of the fixation (Fig. 1). Another eight patients with implant failures underwent conservative treatment because they were asymptomatic (Fig. 2).

In all patients, the average of PSD decreased obviously, from 37 mm to 5.3 mm immediately after surgery. However, compared with postoperative immediate PSD, PSD increased continuously until 6 months after surgery (Table 1).

### Single-plating versus double-plating

Twenty-two patients underwent single plating, and six patients underwent double plating for pubic symphysis diastasis. We compared the results between the single plating group and the double plating group (Table 2). There was no significant difference between the two groups regarding the PSD at the time of the initial injury, immediately postoperatively, at 3 months postoperatively, at 6 months postoperatively, or 12 months postoperatively. Moreover, the differences in implant failure (screw loosening or plate breakage), loss of reduction, initial injured PSD, length of stay in the hospital and the Majeed score were not significant between the two groups. Two patients in the double-plating group developed infections, and this difference was significant (*P = *0.04). After surgical debridement and antibiotic treatment, the infections subsided without residual complications.

**Table 2:** Results of single-plating and double-plating treatments

Parameter	Single plating (*n* = 22)	Double plating (*n* = 6)	*P* value
Initial injured PSD (mm)	37.4 ± 2.25	35.5 ± 13.4	0.846^†^
Postoperative PSD (mm)			
Immediately	5.3 ± 2.0	5.6 ± 2.3	0.711^†^
3 months	7.6 ± 4.0^a^	8.3 ± 3.7^d^	0.574^†^
6 months	8.5 ± 5.3^b^	9.0 ± 3.9 ^e^	0.848^†^
12 months	8.6 ± 5.5^c^	9.1 ± 4.1^f^	0.872^†^
Length of stay (day)	9.5 ± 3.3	9.7 ± 4.2	0.919^†^
No. of implant failures			
Screw loosening	3 (13.7%)	1 (16.7%)	0.643^‡^
Plate breakage	4 (18.2%)	1 (16.7%)	0.715^‡^
No. with loss of reduction	2 (9.1%)	0	0.611^‡^
No. of infections	0	2 (33.3%)	0.040^‡^
Majeed score at final follow-up	79.0 ± 7.1	78.2 ± 4.7	0.737^‡^

Continuous data are expressed as the mean ± standard deviation; categorical data are presented as numbers (percentages)

*PSD* pubic symphysis distance

^a^*P* = 0.001, compared with immediately postoperative PSD

^b^*P* = 0.031, compared with 3-month PSD

^c^*P* = 0.21, compared with 6-month PSD

^d^*P* = 0.023, compared with immediately postoperative PSD

^e^*P* = 0.175, compared with 3-month PSD

^f^*P* = 0.363, compared with 6-month PSD

^a,b,c,d,e,f^computed by paired *t* test

^†^Independent *t* test

^‡^Chi-square test

In addition, we sequentially compared the difference in the PSDs immediately and at 3 months, 6 months, and 12 months postoperatively in each group. In the single-plating group, there were significant differences in the PSD between the immediate findings versus the findings 3 months postoperatively (*P = *0.001) and between the findings at 3 months versus 6 months postoperatively (*P = *0.031). However, there was no significant difference between the findings at 6 months and 12 months postoperatively (*P = *0.21). In the double-plating group, a significant difference was only observed between the values immediately evaluated versus those at 3 months postoperatively (*P = *0.023). No significant difference was observed after 3 months.

### Implant failure versus nonfailure

To further analyze the PSD after implant failure, we divided the patients into two groups (Table 3). One group was the implant failure group, with nine patients, and the other was the nonfailure group, with ninenteen patients. A significant male predominance was observed in the implant failure group (*P = *0.024). There was no significant difference regarding BMI, age, combined posterior fixation, or initial injured PSD between the two groups. The PSD was significantly greater in the implant-failure group than in the non-failure group during the follow-up period at 3 months, 6 months, and 12 months.

**Table 3:** Results of implant failure and nonfailure

Parameter	Implant failure (*n* = 9)	Nonfailure (*n* = 19)	*P* value
Male/female	8/1	8/11	0.024^*^
Age (year)	32.4 ± 20.5	38.2 ± 19.2	0.482^†^
BMI (kg/m^2^)	27.1 ± 3.2	25.5 ± 2.5	0.059^†^
No. of combined posterior fixation	5 (55.6%)	12 (63.2%)	0.507^*^
Initial injured PSD (mm)	35.2 ± 17.1	37.8 ± 22.6	0.381^†^
Postoperative PSD (mm)			
Immediately	6.2 ± 2.1	5.0 ± 1.9	0.066 ^†^
3 months	11.0 ± 4.4^a^	6.4 ± 2.6^d^	0.007^†^
6 months	13.1 ± 6.0^b^	6.5 ± 2.5^e^	0.005^†^
12 months	13.4 ± 6.3^c^	6.7 ± 2.7^f^	0.006^†^
Majeed score at final follow-up	80.1 ± 8.3	78.2 ± 5.9	0.783^‡^
No of intense pain	0	0	
No of same job	2 (22.2%)	7 (36.8%)	0.439 *
No of walking without aids	7 (77.8%)	17 (89.5%)	0.409 *

Continue data are expressed as the mean ± standard deviation; categorical data are presented as numbers (percentages)

*BMI* body mass index, *PSD* pubic symphysis distance

^a^*P* = 0.004, compared with immediately postoperative PSD

^b^*P* = 0.034, compared with 3-month PSD

^c^*P* = 0.081, compared with 6-month PSD

^d^*P* < 0.001, compared with immediately postoperative PSD

^e^*P* = 0.083, compared with 3-month PSD

^f^*P* = 0.084, compared with 6-month PSD

^a,b,c,d,e,f^computed by paired t test

^*^Chi-square test

^†^Independent *t* test

^‡^Mann–Whitney *U* test

The authors also sequentially compared the difference in PSD immediately, at 3 months, 6 months, and 12 months postoperatively in each group. In the implant failure group, a significant difference in the PSD existed between the values measured immediately and those measured at 3 months postoperatively (*P = *0.004) and between the values measured at 3 months and 6 months postoperatively (*P = *0.034). However, no significant difference was revealed in the PDS at 12 months compared to the values taken at 6 months postoperatively (*P = *0.081). In the non-failure group, a significant difference in the PSD was only observed in the values taken at 3 months (*P* < 0.001) compared with the values that were immediately taken postoperatively. After 3 months, no significant difference was observed in the PSD. Finally, there was no significant difference in the Majeed score between the two groups (*P = *0.783). Further analysis of the individual items of the Majeed score revealed no significant differences in the number of patients between the two groups in terms of walking without assistance and returning to the patient’s previous occupation (*P = *0.409 and 0.439). No patients exhibited intense pain at the final follow-up.

## Discussion

The most common management has been open reduction and internal fixation with plating across the disruption symphysis [14, 20, 21]. Loss of the reduction with recurrent diastasis was common after anterior fixation and was reported in 6 of 49 patients by Putnis et al. and in 84 of 127 patients by Collinge et al. [5, 15]. Eastman et al. demonstrated that the average additional symphyseal displacement was 2.6 mm in all patients [17]. In our study, there was a tendency for the gradual widening of the pubic symphysis gap after surgery. A significant increase in the PSD after surgical plating was observed within 6 months postoperatively, especially in the group with single plating or implant failure. However, the PSD was statistically stable from the 6th postoperative month onward.

A high implant failure rate is common for pubic diastasis plating. Pradeep et al. showed that 13 of the 46 patients (28%) in that study had evidence of radiological failure at 31 weeks, and a revision was performed in only one (2%) patient [10]. Morris et al. reported that hardware failure occurred in 43% of the patients and that the revision rate was only 3% [14]. Collinge et al. found that 95 of the 127 patients (75%) in their study had implant failure; of these, only one patient (0.7%) required revision surgery [15]. Putnis et al. reported 15 of 49 patients with a broken or mobile internal metalwork, and four patients required revision surgery [5]. In our study, nine patients (32%) experienced implant failure during the follow-up, but only one (3.6%) required revision surgery. A high implant failure rate with a relatively low revision rate seems to be a special feature of plating for traumatic pubic symphysis diastasis.

This phenomenon could be explained via a biomechanical theory. Anatomically, the pubic symphysis is a fibrocartilaginous joint consisting of a disc sandwiched between the articular surfaces of the pubic bones. It is capable of a small amount of movement under physiological conditions, including up to a 2-mm shift and 1-degree rotation in most adults [22]. A greater degree of motion is especially observed in multiparous females. A rigid fixation of the pubic symphysis restricts the normal physiological movement. Cavalcanti Kußmaul et al. performed a biomechanical study, which confirmed that symphyseal plating as a rigid osteosynthesis almost entirely compromised the physiological mobility of the symphyseal joint [12]. In the long run, physiological micromotion may stress the metalwork of the symphysis, resulting in a gradual increase in the symphyseal space and implant failure, such as screw loosening or plate breakage. In this study, all plate breakage occurred at the symphysis junctions. This can be explained by the fatigue of the metal caused by repeated stress loads. Therefore, the aim of symphysis plating was not to achieve absolute rigid stability but to provide relative stability, so the surrounding tissue of the injured symphysis has the chance to heal. Once the surrounding soft tissue and articular structure healed, the injured pubic symphysis would stabilize, and the PSD remains stable. Similar theory was described in the study by Cavalcanti Kußmaul et al. [12], which presented that a semi-rigid suture provided statistically sufficient biomechanical stability while maintaining a minimum of symphyseal movement, consequently allowing ligamental healing of the injured joint without iatrogenic arthrodesis.

On average, the PSD was mostly stable within 6 months. The timing for the PSD to stabilize in the double-plating group was 3 months and that in the single-plating group was 6 months. In the implant-failure group, the timing for the stability of the PSD was 6 months, which is longer than that of the nonfailure group, which was 3 months. However, the functional outcome at the final follow-up (at least 12 months) was similar between the implant failure and nonfailure groups. No patients had intense pain at the final follow-up, and most patients (77.8% in the failure group and 89.5% in the non-failure group) could walk without aids. This result can be interpreted by the fact that the surrounding soft tissue had healed and ultimately provided sufficient stability. However, many patients changed their occupations or switched to lighter tasks in their current occupations. Only 22.2% in the failure group and 36.8% in the non-failure group kept the same occupation. This may be related to the healed or scarred tissue around the injured pubic symphysis that might only withstand daily activities but not heavy loads.

On the topic of revisions after implant failure, Eastman et al. reported 39 implant failures in 126 disrupted pubic symphyses that were treated with plating. Only two patients with premature postoperative implant failure underwent revision surgeries, and none of the other remaining acute or subacute failures required revision surgery [17]. In this study, only one patient with an implant failure with loss of reduction underwent revision surgery. This case also involved a premature postoperative failure, which was observed immediately during the patient’s hospitalization. Failure that occurs too early with loss of reduction does not provide enough time for the surrounding tissues to heal, so a revision surgery should be considered in these cases.

Regarding the fixation methods, Morris et al. stated that the rate of anterior fixation failure was not related to the type of plate in 148 cases [14]. Aggarwal et al. reported similar outcomes between isolated anterior plating and in combination with perpendicular plating in 13 patients with APC II [23]. MacAvoy et al. reported that single anterior plating for the pubic symphysis had biomechanical properties that were similar to those of two plates in the pelvis with isolated rotational instability [24]. We had similar findings as the above studies. Implant failure was observed in both single plating or double plating, and there were no significant differences between the two methods.

The postoperative PSD during the follow-up was not significantly different between the two methods, although double plating can allow for the injured pubic symphysis to achieve a stable status at 3 months, which is 3 months earlier than single plating. Both infectious complications occurred in patients who had double plating. This may be caused by more soft tissue tripping and dissection during surgery with double plating. However, the final clinical outcome was not significantly different between the two methods. Considering the higher risk of infection after double plating and the similar outcomes between single and double plating, the regular use of double plating may not be required.

The relationship between the symptoms and implant failure remains unclear. Morris et al. described that the majority of patients with hardware failure were asymptomatic [14]. Giannoudis et al. described the postoperative symptoms, including suprapubic pain, neurogenic impotence, and dyspareunia, but none of these symptoms were related to the implant status [3]. The average Majeed score of the implant failure group was slightly higher than that of the nonfailure group, although the difference was not significant (*P = *0.783). The reason for this outcome may be that the implant failure allowed the pubic symphysis to regain a normal physiological micromotion, and this may, in fact, help patients feel more comfortable and improve function.

Although a high incidence of implant failure has been reported, only a few studies have reported the risk factors for implant failure. Based on our results, there were no significant differences in the initial injured PSD, the postoperative PSD, age, the combined posterior fixation or BMI between the implant failure and nonfailure groups. Only male sex had a positive correlation with implant failure. The reason may be that most males engaged in more physical activity than females. This higher level of activity increases the load over the pubic symphysis, which leads to implant failure.

Posterior fixation is important for an unstable posterior pelvic ring injury. When the posterior ring is unstable, as long as it is properly fixed, it will not affect the implant failure of the anterior symphysis. Regarding the fixation of the unstable posterior ring, Cavalcanti Kußmaul et al. reported a biomechanical study which compared 4 different fixations in unstable posterior ring fracture and presented that all methods can achieve stable fixation without significant difference [25].

Some limitations existed in our study. This was a retrospective study, and a small patient sample size was included in our study. Some patients were lost to follow-up and were excluded. The small sample size might have led to the limited power of the study. The follow-up duration was also variable in our cases. Large multicenter studies may be needed to validate the findings of the current study.

In conclusion, gradually increasing the symphyseal distance with a high possibility of implant failure may be a distinguishing feature of traumatic pubic symphysis diastasis fixation. The postoperative symphysis distance becomes stable after 6 months, even after implant failure. The PSD does not seem to be an important postoperative parameter for pubic symphysis diastasis unless a premature postoperative failure occurs. Most patients with implant failure and loss of reduction were asymptomatic and did not require revision surgery. Despite the high rate of radiographic failure, most patients recovered with a good clinical function at the final follow-up. The radiographic outcomes, such as an increased symphysis distance, screw loosening, and plate breakage, did not affect the clinical functional outcomes.
